# Dietary Alpha-Ketoglutarate Partially Abolishes Adverse Changes in the Small Intestine after Gastric Bypass Surgery in a Rat Model

**DOI:** 10.3390/nu14102062

**Published:** 2022-05-14

**Authors:** Paulina Iwaniak, Ewa Tomaszewska, Siemowit Muszyński, Marta Marszałek-Grabska, Stefan Grzegorz Pierzynowski, Piotr Dobrowolski

**Affiliations:** 1 Department of Experimental and Clinical Pharmacology, Medical University, ul. Jaczewskiego 8b, 20-090 Lublin, Poland; dr.iwaniak@gmail.com (P.I.); marta.marszalek-grabska@umlub.pl (M.M.-G.); 2 Department of Animal Physiology, Faculty of Veterinary Medicine, University of Life Sciences in Lublin, Akademicka st. 12, 20-950 Lublin, Poland; ewarst@interia.pl; 3 Department of Biophysics, Faculty of Environmental Biology, University of Life Sciences in Lublin, Akademicka st. 13, 20-950 Lublin, Poland; siemowit.muszynski@up.lublin.pl; 4 Department of Biology, Lund University, Sölvegatan 37, 22362 Lund, Sweden; stefan.pierzynowski@biol.lu.se; 5 Department of Medical Biology, Institute of Rural Health, 20-950 Lublin, Poland; 6 Department of Functional Anatomy and Cytobiology, Faculty of Biology and Biotechnology, Institute of Biological Sciences, Maria Curie-Sklodowska University, Akademicka st. 19, 20-033 Lublin, Poland

**Keywords:** gastric bypass, duodenum, jejunum, histomorphometry, alpha-ketoglutaric acid

## Abstract

Alpha-ketoglutarate (AKG) is one of the key metabolites that play a crucial role in cellular energy metabolism. Bariatric surgery is a life-saving procedure, but it carries many gastrointestinal side effects. The present study investigated the beneficial effects of dietary AKG on the structure, integrity, and absorption surface of the small intestine after bariatric surgery. Male 7-week-old Sprague Dowley rats underwent gastric bypass surgery, after which they received AKG, 0.2 g/kg body weight/day, administered in drinking water for 6 weeks. Changes in small intestinal morphology, including histomorphometric parameters of enteric plexuses, immunolocalization of claudin 3, MarvelD3, occludin and zonula ocludens 1 in the intestinal mucosa, and selected hormones, were evaluated. Proliferation, mucosal and submucosal thickness, number of intestinal villi and Paneth cells, and depth of crypts were increased; however, crypt activity, the absorption surface, the expression of claudin 3, MarvelD3, occludin and zonula ocludens 1 in the intestinal epithelium were decreased after gastric bypass surgery. Alpha-ketoglutarate supplementation partially improved intestinal structural parameters and epithelial integrity in rats undergoing this surgical procedure. Dietary AKG can abolish adverse functional changes in the intestinal mucosa, enteric nervous system, hormonal response, and maintenance of the intestinal barrier that occurred after gastric bypass surgery.

## 1. Introduction

Bariatric surgery is causal and a life-saving procedure. According to numerous recent studies, these types of surgical interventions can help people lose weight and reduce the risk of obesity-related diseases, such as hypertension, ischemic heart disease, stroke, diabetes, gastrointestinal disease, reproductive dysfunction, and cancer [1,2,3]. Unfortunately, although bariatric surgery is one of the most effective therapies, this type of surgery often causes complications that may be related to decreased absorption, impaired intestinal motility, or adverse changes in the structure of the intestines and microbiota [4,5,6]. Other surgical procedures, such as fundectomy, gastrectomy, pancreatoduodenectomy, or partial duodenectomy, necessary in the stomach or duodenal region due to cancer or ulceration, also cause many side effects, similar to those mentioned above, and even more long lasting, such as osteoporosis [7,8,9].There is currently extensive research into the potential use of functional foods, nutraceuticals, and food additives, such as AKG, as supplements in the prevention of lifestyle diseases [10,11,12]. The mechanisms underlying this metabolic improvement, especially one that is independent of body weight, are not fully understood. Alpha-ketoglutaric acid (AKG, 2-ketoglutaric acid, 2-oxoglutaric acid, 2-oxoglutamate or 2-oxopentanedioic acid) is known to play a key role in cellular energy metabolism and is an intermediate in the tricarboxylic acid cycle (TCA, Krebs cycle), which is essential for the oxidation of fatty acids, amino acids, and glucose [13,14]. As a precursor to glutamate and glutamine synthesis in many tissues, AKG bridges the gap between carbohydrate and nitrogen metabolism [15,16,17]. AKG content in foods varies widely, and AKG in the diet is an important source of energy for gastrointestinal epithelial cells [18]. Recent studies point to its functions, including modulating the immune response and intestinal homeostasis [19]. AKG supplementation exhibits potent antiproliferative activity in colon adenocarcinoma cells [20], alleviates mucosal damage, increases the absorptive function of the small intestine in lipopolysaccharide (LPS)-challenged piglets [21], and improves colitis-associated colorectal cancer and gut microbiota in azoxymethane (AOM)/dextran sulfate sodium (DSS)-induced mice. Unlike glutamine, AKG has high stability in aqueous solutions and is well tolerated by the body, which is very important when administered orally [16,22,23,24,25,26]. The global increase in obesity prevalence and its comorbidities has motivated research on the impact of bariatric surgery and its postoperative complications on long-term outcome. The main objective of the present study was to test the hypothesis that dietary AKG supplementation has a beneficial effect on intestinal epithelial and intestinal barrier restoration after gastric bypass surgery, without affecting the body weight of rats undergoing this procedure. The results obtained in this study may support new therapeutic/prophylactic approaches that are needed to reduce the negative long-term effects of gastric bypass surgery in clinical practice.

## 2. Materials and Methods

### 2.1. Animal Breeding and Experimental Design

All procedures performed using animals were approved by the local Ethics Committee of Lund University, Sweden (M143/06), and were performed in accordance with the Guiding Principles for Research Involving Animals.

In this study, 24 seven-week-old male Sprague Dowley rats with an average body weight of 123 g ± 15 g were used. The animals were kept individually in standard rearing conditions (in Macrolon IV cages, Macrolon^®^) with controlled temperature and humidity and 12 h day/night cycle. The rats had free access to water and standard feed for laboratory rodents (Lactamin, Vadstena, Sweden). The experiment lasted for 6 weeks. At the beginning and end of the experiment, the animals were weighed to the nearest 0.01 g using an electronic balance. The animals were divided into four experimental groups (*n* = 6). Two groups were sham operated and another two were subjected to gastric bypass surgery. Subcutaneous injections with Stresnil and Ketaral solutions in physiological saline, in doses of 0.1 and 0.6 mL/kg body weight, respectively, were used for anesthesia. The control group (C) comprised rats submitted to sham surgery, in which the skin, abdominal muscles, and peritoneum were cut in the middle line. Next, the stomach and small intestine were moved, the abdominal wall was stitched. In the experimental group (O), a gastric bypass operation was performed. Briefly, the esophagus, just before the cardia, was cut off of the stomach and joined to the duodenum one centimeter after pylorus. The cardia was immediately stitched to avoid any leakages. The stomach was left intact in the abdominal cavity. The next group (O + AKG) was subjected to gastric bypass (as above) and received a solution of alpha-ketoglutarate (0.2 g/kg/body mass/day) in the drinking water. The last group (AKG) was subjected to a sham surgery and AKG was administered in drinking water (the sodium salt of AKG was administered at a dose of 14.6 g/L). The concentration and duration of AKG administration were based on previous studies, but the dose was reduced because of the baseline age of the rats, which were fully mature and not growing [27,28,29].

### 2.2. Histology Preparation and Histomorphometric Analysis

At the end of the experiment, all animals were sacrificed, and samples of small intestine segments were taken from each animal and subjected to histology. Briefly, 15 mm long segments of the duodenum (20 mm below the stomach) and jejunum (from the middle portion of the small intestine) were fixed in 4% buffered formaldehyde (pH 7.0) for 24 h and rinsed in running water for 4 h. After dehydration in graded ethanol solutions and clearing in xylene, the samples were embedded in paraffin. Then, 4 µm thick cross sections (with a 20 µm interval after each four-slice section) were cut with a microtome (Microm HM 360, Microm, Walldorf, Germany) from every sample of the small intestine. The sections were stained with Goldner’s and Masson’s trichrome methods to differentiate the small intestine wall layers and for planimetric measurements. Hoechst + Eosin staining was used to identify apoptotic cells, and Picro Sirius Red staining (Sirius Red and picric acid, PSR) was carried out to localize fine and coarse collagen fibers [28,30]. Microscopic images were taken for each slide using a confocal microscope (AXIOVERT 200 M, Carl Zeiss, Jena, Germany) and polarizing microscope (Olympus BX63, Tokyo, Japan) for PSR staining using the method described previously [28,31]. Microscopic images of each small intestine segment examined were further analyzed histomorphometrically using graphical analysis software (ImageJ 1.53, National Institutes of Health, Bethesda, MD, USA; accessible at: http://rsb.info.nih.gov/ij/index.html accessed on August 2021). The following parameters were analyzed: the thickness of the inner and outer muscle layer, mucosa and submucosa thickness, crypt depth (defined as the depth of the invagination between adjacent villi from the bottom of the crypt to the base of the villus) and width (measured in the middle of the crypt depth), the height of villi (from the tip of the villus to the villus–crypt junction) and its width (measured in the middle of the villus height), the total number of crypts (opened plus closed crypts), the number of opened crypts (showing mitoses, having an open internal space and access to the intestinal lumen) and closed crypts (not showing mitoses and having a closed lumen), the number of villi (total, intact and damaged) per millimeter of mucosa, height of the villi epithelium, number of proliferating cells per mm of crypts epithelium, the number of enterocytes and Goblet cells per 100 μm of villi epithelium, the number of Paneth’s cells per 10 crypts, number of apoptotic cells/mm^2^ of tissue [32]. The small intestinal absorptive surface was also determined according to Kisielinski [33]. The fractal dimension of the intestinal villi, the surface of the villi epithelium, shape of the lacteal space and the intestinal mucosa. The morphology of the nerve plexuses of the enteric nervous system was studied in the submucosa and muscle membrane. The characteristics of Miessner’s and Auerbach’s plexuses, i.e., mean number, shape, and surface area were analyzed as well in both the duodenum and jejunum part of the small intestine [32].

### 2.3. Immunohistochemical Analysis

The tight junction (TJ) proteins (claudin 3, Marvel D3 (MD3), occludin, zonula occludens 1 (Zo-1) and the cellular proliferation marker protein Ki-67) were evaluated according to the modified protocol previously described [34]. Immunohistochemical reactions were performed on deparaffinized sections of the small intestine. To reduce non-specific background staining due to endogenous peroxidase, slides were incubated in hydrogen peroxide (3% hydrogen peroxide in deionized water) for 10 min. The sections were washed 2 times in PBS buffer. Heat-induced epitope retrieval was performed in sodium citrate buffer (10 mM sodium citrate, 0.05% Tween 20, pH 6.0) using a pressure cooker, Rapid Cook (Morphy Richards, Swinton, UK). The sections were cooled to room temperature and washed 2 times in PBS buffer. Sections were incubated in pre-antibody blocking solution (UltraCruse^®^ Blocking Reagent, Santa Cruz Biotechnology, Inc., Dallas, TX, USA) for 5 min at room temperature. The sections were washed 2 times in PBS buffer. The sections were then incubated with primary antibodies for one hour at room temperature in a humid chamber. All primary antibodies were rat specific (rabbit as a host): anti-Ki-67 (AB16667, monoclonal, Abcam, Cambridge, UK, 1:200); anti-claudin 3 (AB15102, polyclonal, Abcam, Cambridge, Great Britain 1:100); MarvelD3 (PA5-42629, polyclonal, Invitrogen, Thermo Fisher Scientific, Waltham, MA, USA, dilution 1:100); occludin (13409-1-AP, polyclonal, Proteintech, Wuhan, China, 1:100), and anti-Zo-1 (zonula occludens 1) (orb11587, Biorbyt, St. Louis, MO, USA, dilution 1:100) were used. The sections were washed 2 times in PBS buffer. Post-antibody blocking solution (DPVB110HRP, BrightVision, two-step detection system Goat Anti-Mouse/Rabbit HRP) was applied for 15 min and then sections were washed 2 times in PBS buffer. The sections were then incubated for 30 min with Poly-HRP-Goat Anti-Mouse/Rabbit IgG (DPVB110HRP, BrightVision, two-step detection system Goat Anti-Mouse/Rabbit HRP) at room temperature. After washing sections 2 times in PBS buffer the reaction was visualized with the use of DAB+ (3,3′-diaminobenzidine chromogen solution and imidazol-HCl buffer, pH 7.5, containing hydrogen peroxide and an antibacterial agent) as a dye (DakoCytomation, DakoCytomation Denmark A/S, Glostrup, Denmark) for 15 min at room temperature. In the control reaction, the first antibody was replaced with PBS. Counterstaining was performed with Mayer’s hematoxylin (Sigma-Aldrich, St. Louis, MO, USA). Four determinations were made for each specimen, which were photographed for further analysis. Microscopic images of each small intestine segment examined were further analyzed histomorphometrically using graphical analysis software (ImageJ 1.53, National Institutes of Health, Bethesda, MD, USA; accessible at: http://rsb.info.nih.gov/ij/index.html accessed on August 2021). The integrated intensity of immunoreaction was measured by comparing pixel brightness values adjusted for DAB staining color detection, using IHC Tool Box color analysis with H-DAB model and inverted 8-bit grayscale, such that a higher pixel value reflects a higher immunoreaction intensity (darkness of the assessed elements).

### 2.4. Statistical Analysis

The results are expressed as means ± SEM. One-way ANOVA followed by Tukey’s multiple comparison posttests, the W. Shapiro–Wilk test and the Brown–Forsythe test was performed using STATISTICA (data analysis software system), version 12. StatSoft, Inc., Tulsa, OK, USA (2014). In reasonable cases, the Kruskal–Wallis and median test was applied. The statistical model presented below was used to analyze selected parameters.

x_ij_ = µ + α_i_ + β_j_ + (αβ)_ij_ + ε_ijk_ (1)

where: x_ij_—observation (intestinal parameter), i—the level of the first factor (diet: no supplementation, supplementation with AKG), j—level of the second operation factor (use of the bariatric surgery), or not, k—number of measurements, μ—constant, general mean, α_i_—main effect of the first factor, β_j_—main effect of the second factor, (αβ)_ij_—interaction effect of main factors, ε_ijk_—random error. Values of *p* < 0.05 were considered statistically significant.

## 3. Results

### 3.1. Body Weight

**Figure 1:**
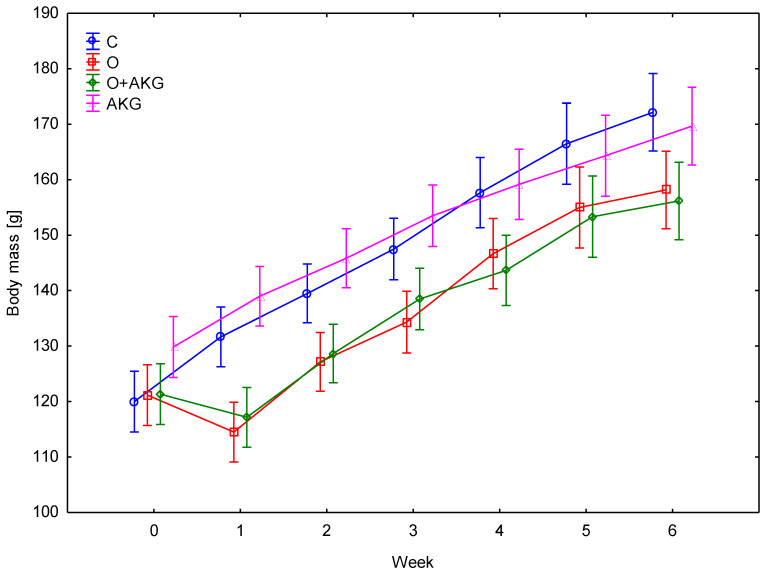
Effect of bariatric surgery and administration of alpha-ketoglutarate (AKG) on body weight in rats. C—control, O—gastric bypass surgery, O + AKG—gastric bypass surgery and administration of alpha-ketoglutarate, AKG—administration of alpha-ketoglutarate. Alpha-ketoglutarate (0.2 g/kg/body mass/day) was administered in the drinking water.

Rats in the control and experimental groups had similar initial weights, averaging 123 g ± 15 g. AKG supplementation did not cause differences in animal body weights. However, a significant effect of surgery was observed in the first week after gastric bypass, when the operated rats showed a 15% reduction in weight gain (*p* = 0.012) compared to the sham-operated groups. In subsequent weeks, there was no difference in the rate of weight gain between groups (Figure 1).

### 3.2. Histomorphometry of the Small Intestine

**Figure 2:**
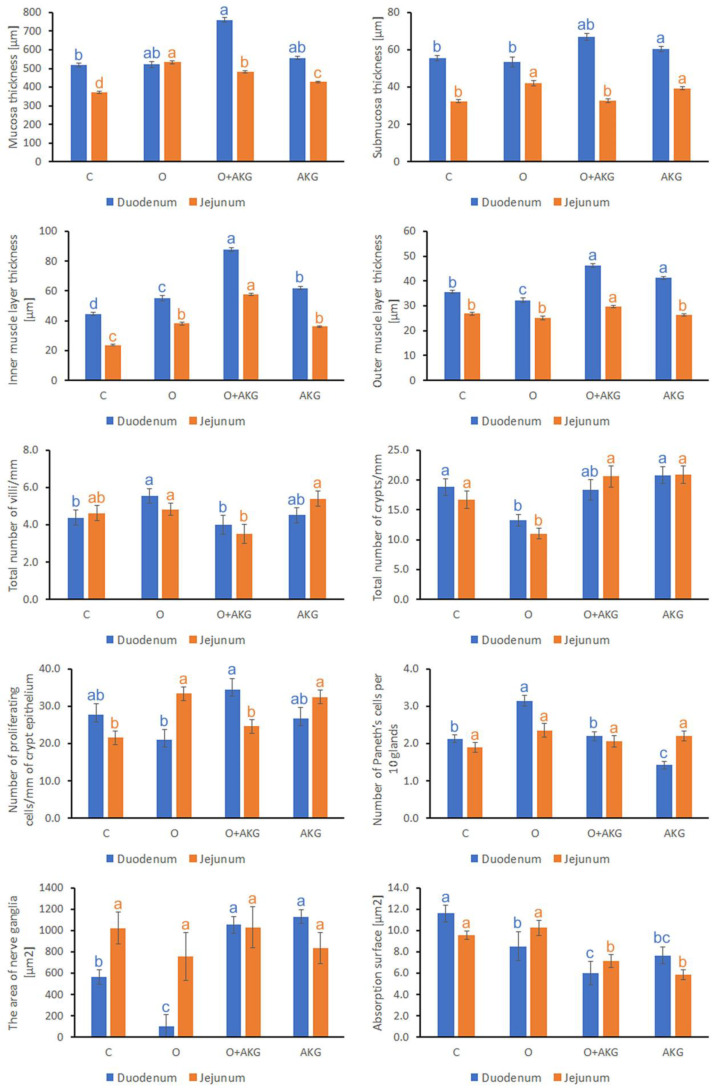
Effects of bariatric surgery and alpha-ketoglutarate (AKG) administration on selected structural parameters of the duodenum and jejunum in rats. C—control, O—gastric bypass surgery, O + AKG—gastric bypass surgery and administration of alpha-ketoglutarate, AKG—administration of alpha-ketoglutarate. Alpha-ketoglutarate (0.2 g/kg/body mass/day) was administered in the drinking water. Different letters above the bars indicate significant differences at *p* < 0.05, and colors are used to indicate the corresponding intestinal segments.

Surgical intervention resulted in significant differences in the histological structure of the duodenal and jejunal walls for almost all parameters studied, as shown in Figure 2 and Table 1. Gastric bypass surgery and AKG supplementation increased mucosal (44%, *p* < 0.001) and submucosal (30%, *p* < 0.001) thickness in the jejunum and duodenum, respectively. Furthermore, AKG administration after surgery increased muscle thickness in both jejunum and duodenum (Figure 2). Compared with the control group, several intestinal indices were significantly reduced in the duodenum of rats after gastric bypass surgery, namely: intestinal villi length (31%, *p* = 0.028), number of intestinal crypts (30%, *p* = 0.047), including the number of active crypts (42%, *p* = 0.025) (Figure 2, Table 1 and Table S1). Like the duodenum, the total number of crypts in the jejunum after gastric surgery was 34% lower (*p* = 0.048) (Figure 2). More crypts were observed in the sham-operated, AKG-supplemented group, but these differences were not statistically significant (*p* < 0.05) (Figure 2). In addition, the depth of the intestinal crypts was significantly greater in all groups compared with the control group. Gastric bypass surgery increased crypt depth by 65% (*p* < 0.001) in the duodenum and by 147% (*p* < 0.001) in the jejunum (Table 1). Gastric bypass resulted in a 26% increase in the number of villi in the duodenum (*p* = 0.044). The opposite result was observed in rats after surgery and AKG supplementation (Figure 2). The total number of intestinal villi in this group was reduced, but they were wider than in rats in the operated group, that is, by 188% in the jejunum (*p* < 0.001) and by 162% in the duodenum (*p* < 0.001) (Table 1). Epithelial height increased in the O + AKG group by 11% (*p* < 0.001) in the duodenum and 10% (*p* < 0.001) in the jejunum compared to respective controls; no other changes were observed (Table 1).

Gastric bypass surgery did not change the number of enterocytes in the duodenum (*p* = 0.867) and in the jejunum (*p* = 0.565). In contrast, AKG supplementation reduced the number of enterocytes per 100 μm of villi epithelium by 35% (*p* < 0.001) in the jejunum and 19% (*p* < 0.001) in the duodenum. In addition, a 25% (*p* < 0.001) decrease in the number of enterocytes was observed in the operated and supplemented groups, but only in the jejunum (Table 1). The number of Paneth cells increased by 45% (*p* = 0.004) after surgery in the duodenum, whereas AKG administration in sham-operated rats decreased their number by 33% (*p* = 0.004). The differences in Paneth cell numbers observed in the jejunum were not significant (*p* > 0.05) (Figure 2).

Evaluation of the number of proliferating cells in the intestinal glands showed differential effects of both AKG and surgery on the selected small intestinal segment. Gastric bypass surgery increased proliferation in the jejunum and supplementation of AKG to operated animals reversed this effect. On the contrary, the opposite situation was observed in the duodenum; however, the effect was not significant compared to control. The greatest increase in proliferating cells was observed in the jejunum after surgery. Their number increased by 54% (*p* < 0.001) compared with the control group. In contrast, in the duodenum, surgery resulted in a 24% decrease in the number of proliferating cells, but this difference was not statistically significant (*p* = 0.394). Approximately 25% (*p* = 0.013) more proliferating cells were observed in the duodenum of animals after surgery and AKG supplementation compared to controls. In addition, AKG supplementation caused a 50% (*p* = 0.010) increase in the number of proliferating cells in the jejunum, with no significant effect in the duodenum (*p* = 0.995) (Figure 2).

Bariatric surgery reduced the number of apoptotic cells in the duodenum by 60% (*p* = 0.001), whereas in the jejunum, the value was similar to control (*p* = 0.501). In the O + AKG group, the number of apoptotic cells was reduced by 81% in the duodenum (*p* < 0.001) and by 13% in the jejunum (*p* = 0.501). The greatest increase in apoptotic cells was observed in the jejunum of AKG-supplemented rats, where their number increased by 154% (*p* < 0.001) compared with the control group. In contrast, a 48% decrease in this parameter was observed in the duodenum (*p* = 0.003) (Table 1).

A significant reduction in absorption area in the small intestine was observed in all groups in both intestinal segments, except in the jejunum in the operated group, compared to the respective controls. Moreover, AKG supplementation further reduced the absorption surface area. A large reduction in absorption area was observed in both intestinal segments, i.e., by 34% (*p* = 0.003) in the duodenum and 39% (*p* = 0.001) in the jejunum (Figure 2).

The area of nerve ganglia was drastically reduced in the duodenum of operated rats, whereas AKG supplementation increased this parameter compared to control, with no significant effects observed in the jejunum (Figure 2).

Neither bariatric surgery nor AKG supplementation had any effect on the number of ganglia in small intestinal nerve plexuses (*p* > 0.05). Analysis of the plexus shape showed no statistically significant differences between the groups, as did other shape descriptors of the other parameters measured (Supplementary Tables S1 and S2).

### 3.3. Biochemical Analysis of Gastrin and CCK

The levels of gastrin and cholecystokinin (CCK) in the blood are presented in Table 2. After the bariatric surgery, the level of gastrin in the blood decreased significantly by 85% (*p* = 0.002). The level of cholecystokinin was also reduced by 43%; however, this was not statistically significant (*p* = 0.156). In the operated and AKG-supplemented group, the level of gastrointestinal hormones decreased by 77% (*p* = 0.001) in the case of gastrin and by 58% (*p* = 0.016) for cholecystokinin. In turn, the difference in the quantity of both hormones in the rats receiving only AKG was significantly reduced, i.e., by 6% (*p* = 0.997) for gastrin and by 11% (*p* = 0.869) for cholecystokinin.

### 3.4. Immunohistochemical Analysis of Selected Proteins of the Small Intestinal Barrier

**Figure 3:**
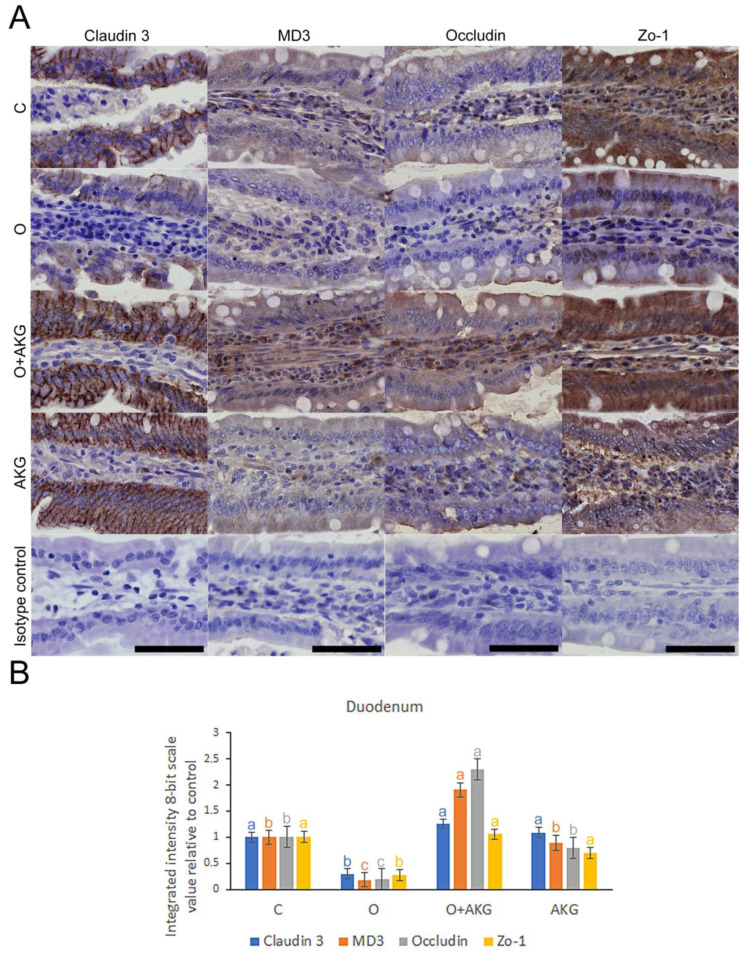
Effects of bariatric surgery and alpha-ketoglutarate (AKG) administration on selected tight junction proteins of the intestinal barrier. (**A**) Representative images of the immunolocalization and distribution of claudin 3, MD3, occludin and Zo-1 in the rat duodenum. C—control group undergoing sham surgery; O—group undergoing gastric bypass surgery; O + AKG—group undergoing gastric bypass surgery and receiving alpha-ketoglutarate (AKG); AKG—group undergoing sham surgery and receiving alpha-ketoglutarate. AKG was administered at a dose of 0.2 g/kg/body weight/day in drinking water. All the scale bars represent 50 µ. (**B**) The bar graph shows the integrated intensity of immunoreactions, measured by comparing pixel brightness values adjusted to DAB staining color detection and inverted 8-bit grayscale, such that a higher pixel value reflects a higher immunoreaction intensity. Different letters above the bars indicate significant differences at *p* < 0.05, and colors are used to indicate the corresponding tight junction protein.

**Figure 4:**
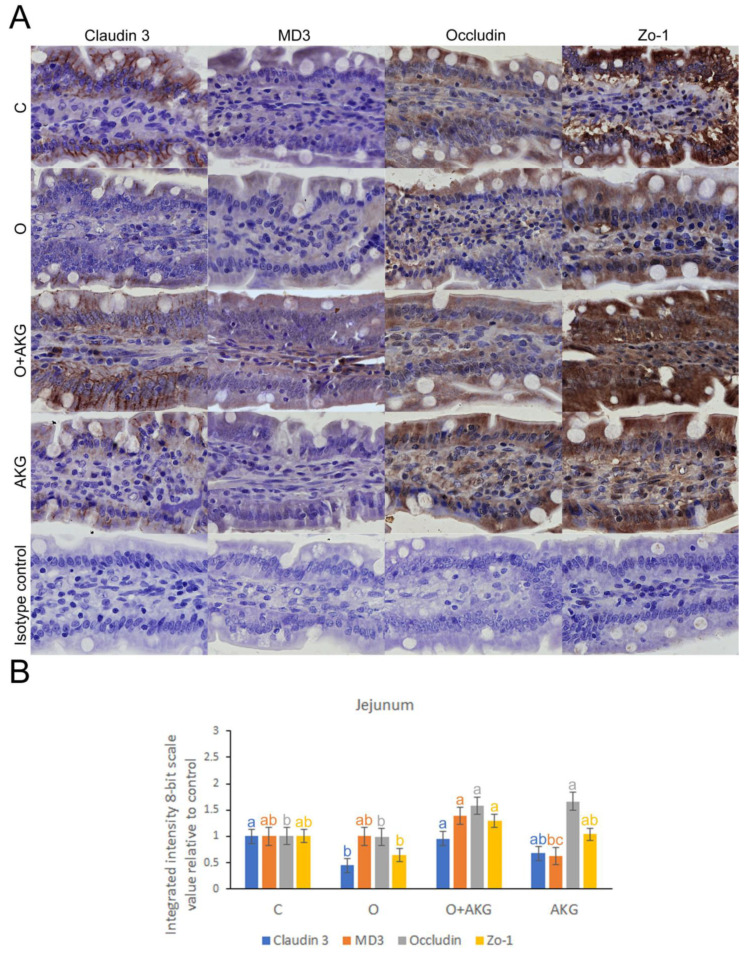
Effects of bariatric surgery and alpha-ketoglutarate (AKG) administration on selected tight junction proteins of the intestinal barrier. (**A**) Representative images of the immunolocalization and distribution of claudin 3, MD3, occludin and Zo-1 in the rat jejunum. C—control group undergoing sham surgery; O—group undergoing gastric bypass surgery; O + AKG—group undergoing gastric bypass surgery and receiving alpha-ketoglutarate (AKG); AKG—group undergoing sham surgery and receiving alpha-ketoglutarate. AKG was administered at a dose of 0.2 g/kg/body weight/day in drinking water. All the scale bars represent 50 µ. (**B**) The bar graph shows the integrated intensity of immunoreactions, measured by comparing pixel brightness values adjusted to DAB staining color detection and inverted 8-bit grayscale, such that a higher pixel value reflects a higher immunoreaction intensity. Different letters above the bars indicate significant differences at *p* < 0.05, and colors are used to indicate the corresponding tight junction protein.

Examples of immunohistochemical detection and spatial distribution of claudin 3, MD3, occludin and Zo-1, as well as the results of integrated intensity of immunoreaction analysis, are shown in Figure 3 and Figure 4. Gastric bypass surgery led to a significant reduction in the intensity of the detection response and spatial distribution of all TJ proteins tested in the duodenum, but only in case of claudin 3 in the jejunum. AKG supplementation abolished this effect in the O + AKG group in both intestinal segments tested.

## 4. Discussion

Surgical treatment, which includes gastric bypass, disrupts the physiology of the digestive system, causing changes in gastrointestinal function, often leading to deficiencies of certain nutrients [35].

The small intestine is not only the terminal organ for digestion and absorption of nutrients from the diet, but it is also crucial for preventing exogenous pathogens from entering the systemic circulation [36,37]. Gut integrity is, therefore, essential for the survival, growth, and health of both animals and humans [38]. Extensive studies in experimental animals, including rats, have shown that a number of stressors, such as infections, inflammation, and surgical interventions, can lead to damage and dysfunction in the intestinal mucosa [21,39]. Therefore, restitution of the intestinal epithelial barrier plays a key role in maintaining gastrointestinal tract homeostasis under stress conditions [40].

Multiple lines of evidence suggest a link between tight junction proteins and epithelial restitution [41]. Tight junctions are the most apical intercellular structures in epithelial and endothelial cells that regulate lateral intercellular permeability and are crucial for epithelial barrier integrity [42]. Thus, to test whether AKG can promote intestinal epithelial restitution under stress injury, the expression and localization of tight junctions in the small intestine were determined by immunohistochemical analysis. We investigated the localization, distribution, and integrated intensity of immunoreaction in representatives of integral transmembrane proteins, such as claudin 3 and occludin, and peripheral membrane adaptor proteins, such as zonula occludens 1, as well as the transmembrane protein MarvelD3, acting as a regulator of epithelial cell proliferation, migration, and survival [43,44]. Bariatric surgery disrupted their primary distribution and subsequently led to intestinal barrier dysfunction. AKG supplementation improved the restoration of these tight junction proteins. These findings support many previous studies that reported that decreased expression of tight junctions can lead to alteration or disruption of the intestinal barrier [45]. Reduced AKG synthesis has recently been demonstrated in IBD patients with increased intestinal permeability associated with altered tight junction expression [46]. Our results showed that bariatric surgery significantly decreased the expression of TJ proteins, as reported in previous studies [47]. As expected, AKG administration increased the expression of TJ proteins in the intestine of operated rats, suggesting that AKG administration enhances the protective and restorative function of some TJ proteins in stress defense after gastric surgery. Recent studies in mammals have shown that AKG used as a dietary supplement can have beneficial effects on both injured and healthy organisms [48,49]. He L. et al. demonstrated that AKG administration can lead to increased intestinal-mucosal mass and improved tight junctions under stress conditions [41]. In addition, AKG administration was shown to positively modulate antioxidant capacity and protein synthesis to improve epithelial restitution in rats under protein deficiency and oxidative stress [48,50].

Gut morphology is a major indicator of intestinal health and also reflects the maturation rate of enterocytes [41]. The height of the villi and the depth of the crypts reflect the number and maturation rate of enterocytes, which affect the ability to absorb and transport nutrients in the intestine [51]. In addition to morphological changes, it also includes adaptations in the nervous system, endocrine system, and nutrient signaling. Seeley et al. hypothesized that changes in neuronal innervation or neuronal activity may result in an increase in enteroendocrine cell number or sensitivity to stimuli and increased nutrient absorption by increasing the number and/or length of villi and/or depth of crypts, as well as stimulation of intracellular signaling processes by increasing nutrient transport or production of digestive products [35]. In the present study, gastric bypass resulted in a decrease in the absorptive surface area in the duodenum and an increase in this parameter in the jejunum. In contrast, the AKG diet caused an increase in the height and width of villi, depth of crypts, and thickness of mucosa in the small intestine of rats undergoing surgery. These results were consistent with previous studies, suggesting that AKG in enterocytes can beneficially regulate the intracellular concentration of endogenous amino acids through the TCA cycle and subsequently affect various signaling pathways, such as AMP-activated protein kinase (AMPK), nuclear factor kappa B (NF-κB), and mammalian target of rapamycin (mTOR) pathways, thereby improving intestinal morphology [41].

AKG supplementation increased villi height in the jejunum, as well as crypt depth and mucosal thickness, in both the duodenum and jejunum. These results suggest that AKG can improve intestinal morphology after bariatric surgery in rats. In the current study, minor morphological changes were observed in the duodenum after bariatric surgery, whereas there was hypertrophy in the jejunum. Such morphological changes have been observed after various surgical procedures involving manipulation of the gastrointestinal tract in rodent models. Li et al. observed that when the stomach was left intact and the upper intestine was bypassed (duodenojejunal bypass), atrophy was observed in the bypassed organ and hyperplasia was observed in the portion of the jejunum exposed to nutrients [52]. Similarly, intestinal proliferation was observed in earlier studies after Roux-en-Y gastric bypass, where significant increases in cell proliferation, intestinal width, villi height, and crypt depth were observed in the terminal gastrointestinal tract and common intestine [53,54]. These results indicate that functional elimination of one part of the gastrointestinal tract may cause a compensatory response in the other parts, involving some form of morphological adaptation. Thus, bypass surgery may cause hypertrophy in the remaining part of the intestine. This hypertrophy is the result of intestinal hyperplasia, which is associated with a higher rate of cell proliferation in the crypts and an increase in crypt depth and villi height, as confirmed by a previous study by McDuffie et al. [55]. In light of these results, the hypertrophy coupled with an increase in cell proliferation and a concomitant decrease in enterocytes observed in jejunum in the current study can be explained by the extent of the surgical procedure performed and further studies are needed to understand the underlying mechanisms.

Data from detailed histomorphometric analyses performed in the present study confirm the effects of AKG supplementation observed in previous studies. However, in this study, AKG did not have any effect on body weight. The significantly increased villi height, crypt depth, mucosal thickness, and crypt and villi width observed in the present study after dietary administration of AKG is consistent with the results of previous studies [21,38,48].

The current study is unique in that it is the first study to compare, in detail, the effects of bariatric surgery, including gastric bypass, on small intestinal development and structure in rats. Furthermore, the results presented here also show, for the first time, an improvement in small intestinal innervation after AKG administration in rats undergoing bariatric surgery. The gastrointestinal tract is innervated by an extensive internal network of ganglion-rich nerve connections, called the enteric nervous system (ENS), which can be divided into two main networks, the myenteric and submucosal plexuses, also known as the Auerbach and Meissner plexuses, respectively [56,57,58]. The ENS is involved in the control of intestinal motility, blood flow, mucosal and secretory transport, as well as endocrine and immune functions. In the present study the parameters of the myenteric (Auerbach) and submucosal (Meissner) plexuses were investigated. Gastric bypass surgery was found to slightly (but not significantly) reduce the number of Auerbach’s ganglia in the duodenum and significantly reduce the size of Meissner’s and Auerbach’s ganglia in the duodenum (Table S1 and Figure 2). In contrast to the operated group, AKG significantly improved innervation and reduced the negative effects of surgery on the small intestine in rats. AKG supplementation significantly increased the area of ganglia, in the duodenum.

The gut is the largest endocrine organ in the body, expressing over 30 gut hormone genes and a wealth of bioactive peptides [59]. As two of the first gastrointestinal peptides discovered, gastrin and CCK play important roles in digestive processes, including gastric acid secretion, pancreatic enzyme release, gallbladder emptying, intestinal motility, and energy homeostasis [60,61]. As classical gut hormones and potent neurotransmitters, gastrin and CCK are widely distributed in the gastrointestinal tract, CNS, and peripheral neurons [61,62]. According to studies, the expression and secretion of hormones in the small intestine depend on systemic factors related to metabolic status, as well as locally acting factors [63]. Changes in the number or density of endocrine cells have been observed after various anastomosis procedures. In the present study, the decrease in serum gastrin and CCK levels after bariatric surgery appears to be a natural response associated with the lack of stimulation of the bypassed stomach (Table 2). Dietary AKG supplementation did not significantly alter CCK secretion, whereas gastrin was higher compared with the surgery group. Because gastric-derived hormones are involved in various activities throughout the GIT, eliminating their effects/functions after gastric bypass surgery may be important not only for GIT function but also for the control of food intake, glycemia, and other activities [64].

Although the study does not focus on body weight, body weight is significantly reduced after bariatric surgery [65]. AKG did not alter body weight gain in operated rats (Figure 1), which would be an important issue with long-term supplementation in practice.

Although many of the beneficial metabolic effects of bariatric surgery can be attributed to weight loss, the rapid changes in most gut hormones, accompanied by improvements in comorbidities, suggest that anatomic and physiologic changes after surgery are also important.

Finally, looking to the future, we discuss the need to translate the wealth of data obtained from animal studies to the clinical setting and, thus, to better understand all the changes in GIT physiology and morphology. Therefore, although the presented study has some limitations, such as the lack of detailed data on serum biochemical parameters and accurate hormonal analysis, we are confident that the results obtained can be clinically helpful to investigate the similarities between rat and human models, including changes in the postoperative intestinal hormone profile and metabolic effects in bariatric patients. We acknowledge that our results represent structural changes in the small intestinal barrier; however, the absorption surface area that was assessed in this study reflects, to some extent, the ability of the intestine to absorb nutrients, and defects in the intestinal barrier reflect a degree of permeability that is widely reported in the literature [43]. At this point, we would like to gain more insight into actual nutrient absorption, but the purpose of this study was to elucidate the potential for structural changes and subsequent prognosis after bariatric surgery and the impact of AKG supplementation. Based on the current promising data on the effects of AKG, further studies are needed to further study this topic and reveal the relationship between the intestinal barrier and hormones, based on more complex additional studies, e.g., hormone receptors, additional markers, and functional studies of permeability and absorption.

## Figures and Tables

**Table 1:** Effects of bariatric surgery and alpha-ketoglutarate (AKG) administration on the histological structure of selected parameters of the duodenum and jejunum of the rat.

Parameter	C	O	O + AKG	AKG
*Duodenum*				
Villi width/µm	119.8 ± 43.4 ^c^	121.6 ± 33.8 ^bc^	313.3 ± 100.6 ^a^	200.9 ± 69.0 ^b^
Villi length/µm	623.8 ± 153.3 ^a^	433.6 ± 51.6 ^c^	572.5 ± 89.8 ^ab^	521.3 ± 79.8 ^b^
Crypt depth/µm	97.3 ± 52.9 ^c^	160.6 ± 28.8 ^bc^	205.4 ± 94.0 ^a^	152.2 ± 38.7 ^b^
Crypt width/µm	44.4 ± 6.8 ^b^	41.7 ± 7.4 ^bc^	48.9 ± 9.0 ^a^	40.9 ± 7.1 ^c^
The height of the villi epithelium [µm]	32.3 ± 0.3 ^b^	32.2 ± 0.5 ^b^	36.0 ± 0.4 ^a^	32.4 ± 0.3 ^b^
Number of enterocytes per 100 μm of villi epithelium	18.6 ± 4.0 ^a^	17.8 ± 4.0^a^	17.8 ± 3.8 ^a^	15.0 ± 3.7 ^b^
Number of apoptotic cells/mm^2^ of tissue	2.4 ± 0.2 ^a^	1.0 ± 0.2 ^bc^	0.5 ± 0.1^c^	1.3 ± 0.2 ^b^
*Jejunum*				
Villi width [µm]	79.3 ± 32.0 ^b^	101.6 ± 23.4 ^b^	228.2 ± 26.9 ^a^	206.8 ± 32.6 ^a^
Villi length [µm]	439.5 ± 70.0 ^b^	486.5 ± 74.7 ^ab^	515.9 ± 113.7 ^a^	395.4 ± 52.9 ^c^
Crypt depth [µm]	66.3 ± 22.8 ^c^	163.7 ± 38.4 ^a^	161.0 ± 40.1 ^a^	128.4 ± 28.7 ^b^
Crypt width [µm]	43.6 ± 8.9 ^a^	40.4 ± 7.8 ^ab^	42.7 ± 7.7 ^a^	38.0 ± 7.1 ^b^
The height of the villi epithelium [µm]	30.8 ± 0.3 ^b^	30.4 ± 0.6 ^b^	33.8 ± 0.4 ^a^	30.7 ± 0.4 ^b^
Number of enterocytes per 100 μm of villi epithelium	24.3 ± 5.4 ^a^	21.6 ± 3.9 ^ab^	18.2 ± 3.2 ^bc^	15.7 ± 4.3 ^c^
Number of apoptotic cells/mm^2^ of tissue	0.7 ± 0.2 ^b^	0.7 ± 0.1 ^b^	0.6 ± 0.1 ^b^	1.8 ± 0.2 ^a^

Data are presented as mean ± SD. Different superscript letters in a row indicate significant differences at *p* < 0.05. C—control group undergoing sham surgery; O—group undergoing gastric bypass surgery; O + AKG—group undergoing gastric bypass surgery and receiving alpha-ketoglutarate (AKG); AKG—group undergoing sham surgery and receiving alpha-ketoglutarate. AKG was administered at a dose of 0.2 g/kg/body weight/day in drinking water.

**Table 2:** Effects of bariatric surgery and alpha-ketoglutarate (AKG) administration on cholecystokinin and gastrin levels in rats.

Parameter	C	O	O + AKG	AKG
Cholecystokinin	6.3 ± 1.9 ^a^	3.6 ± 1.4 ^ab^	2.7 ± 1.1 ^b^	5.6 ± 1.3 ^ab^
Gastrin	61.2 ± 16.1 ^a^	9.3 ± 1.5 ^b^	14.0 ± 2.9 ^b^	57.8 ± 17.6 ^a^

Data are presented as mean ± SD. Different superscript letters in a row indicate significant differences between groups at *p* < 0.05. C—control group undergoing sham surgery; O—group undergoing gastric bypass surgery; O + AKG—group undergoing gastric bypass surgery and receiving alpha-ketoglutarate (AKG); AKG—group undergoing sham surgery and receiving alpha-ketoglutarate. AKG was administered at a dose of 0.2 g/kg/body weight/day in drinking water.

## Data Availability

The data presented in this study are available on request from the corresponding author.

## Supplementary Materials

The following supporting information can be downloaded at: https://www.mdpi.com/article/10.3390/nu14102062/s1, Table S1: The effect of bariatric surgery and alpha-ketoglutarate administration on histomor-phometry of selected parameters of rat duodenum; Table S2: The effect of bariatric surgery and alpha-ketoglutarate administration on histomor-phometry of selected parameters of rat jejunum.

## References

[1] Reilly J.J., Methven E., McDowell Z.C., Hacking B., Alexander D., Stewart L., Kelnar C.J.H. (2003). Health consequences of obesity. Arch. Dis. Child..

[2] Bray G.A. (2004). Medical consequences of obesity. Proc. J. Clin. Endocrinol. Metab..

[3] Birks S., Peeters A., Backholer K., O’Brien P., Brown W. (2012). A systematic review of the impact of weight loss on cancer incidence and mortality. Obes. Rev..

[4] Fu X.-Y., Li Z., Zhang N., Yu H.-T., Wang S.-R., Liu J.-R. (2014). Effects of gastrointestinal motility on obesity. Nutr. Metab..

[5] Nam S.Y. (2017). Obesity-Related Digestive Diseases and Their Pathophysiology. Gut Liver.

[6] Balan H., Costache C., Angelescu G., Popescu L. (2017). Bariatric surgery—A real hope or just fake news?. Arch. Balk. Med. Union.

[7] Seo G.H., Kang H.Y., Choe E.K. (2018). Osteoporosis and fracture after gastrectomy for stomach cancer. Medicine.

[8] Kapoor V.K. (2016). Complications of pancreato-duodenectomy. Rozhl. V Chir. Mesic. Ceskoslovenske Chir. Spol..

[9] Sah B., Zhu Z., Wang X., Yang Q., Chen M., Xiang M., Chen J., Yan M. (2009). Post-operative complications of gastric cancer surgery: Female gender at high risk. Eur. J. Cancer Care.

[10] Nasri H., Baradaran A., Shirzad H., Rafieian-Kopaei M. (2014). New Concepts in Nutraceuticals as Alternative for Pharmaceuticals. Int. J. Prev. Med..

[11] Sachdeva V., Roy A., Bharadvaja N. (2020). Current Prospects of Nutraceuticals: A Review. Curr. Pharm. Biotechnol..

[12] Wan M.L.Y., Ling K.H., El-Nezami H., Wang M.F. (2018). Influence of functional food components on gut health. Crit. Rev. Food Sci. Nutr..

[13] Harrison A.P., Pierzynowski S.G. (2008). Biological effects of 2-oxoglutarate with particular emphasis on the regulation of protein, mineral and lipid absorption/metabolism, muscle performance, kidney function, bone formation and cancerogenesis, all viewed from a healthy ageing perspective state of the art—Review article. J. Physiol. Pharmacol. Off. J. Pol. Physiol. Soc..

[14] Xiao D., Zeng L., Yao K., Kong X., Wu G., Yin Y. (2016). The glutamine-alpha-ketoglutarate (AKG) metabolism and its nutritional implications. Amino Acids.

[15] Owen O.E., Kalhan S., Hanson R.W. (2002). The Key Role of Anaplerosis and Cataplerosis for Citric Acid Cycle Function. J. Biol. Chem..

[16] Junghans P., Derno M., Pierzynowski S., Hennig U., Rudolph P.E., Souffrant W.B. (2006). Intraduodenal infusion of α-ketoglutarate decreases whole body energy expenditure in growing pigs. Clin. Nutr..

[17] Pierzynowski S., Pierzynowska K. (2022). Alpha-ketoglutarate, a key molecule involved in nitrogen circulation in both animals and plants, in the context of human gut microbiota and protein metabolism. Adv. Med. Sci..

[18] Pierzynowski S.G., Sjodin A. (1998). Perspectives of glutamine and its derivatives as feed additives for farm animals. J. Anim. Feed Sci..

[19] Chen S., Bin P., Ren W., Gao W., Liu G., Yin J., Duan J., Li Y., Yao K., Huang R. (2017). Alpha-ketoglutarate (AKG) lowers body weight and affects intestinal innate immunity through influencing intestinal microbiota. Oncotarget.

[20] Rzeski W., Walczak K., Juszczak M., Langner E., PoŻarowski P., Kandefer-Szerszeń M., Pierzynowski S.G. (2012). Alpha-ketoglutarate (AKG) inhibits proliferation of colon adenocarcinoma cells in normoxic conditions. Scand. J. Gastroenterol..

[21] Hou Y., Wang L., Ding B., Liu Y., Zhu H., Liu J., Li Y., Wu X., Yin Y., Wu G. (2010). Dietary α-ketoglutarate supplementation ameliorates intestinal injury in lipopolysaccharide-challenged piglets. Amino Acids.

[22] Buddington R.K., Pajor A., Buddington K.K., Pierzynowski S. (2004). Absorption of α-ketoglutarate by the gastrointestinal tract of pigs. Comp. Biochem. Physiol. Part A Mol. Integr. Physiol..

[23] Śliwa E., Dobrowolski P., Siwicki A.K., Pierzynowski S.G. (2007). Changes of a non-specific defence mechanism in blood serum of piglets induced by prenatal and postnatal administration of α-ketoglutarate. Bull. Vet. Inst. Pulawy.

[24] Śliwa E., Dobrowolski P., Tatara M.R., Pierzynowski S.G. (2007). Alpha-ketoglutarate partially protects newborns from metabolic changes evoked by chronic maternal exposure to glucocorticoids. J. Pre-Clin. Clin. Res..

[25] Filip R., Pierzynowski S.G. (2008). The absorption, tissue distribution and excretion of enteraly administered α-ketoglutarate in rats. J. Anim. Physiol. Anim. Nutr..

[26] Grzesiak P., Słupecka-Ziemilska M., Woliński J. (2016). The biological role of a-ketoglutaric acid in physiological processes and its therapeutic potential. Dev. Period Med..

[27] Dobrowolski P., Tomaszewska E., Radzki R.P., Bienko M., Wydrych J., Zdybel A., Pierzynowski S.G. (2013). Can 2-oxoglutarate prevent changes in bone evoked by omeprazole?. Nutrition.

[28] Dobrowolski P., Tomaszewska E., Kurlak P., Pierzynowski S.G. (2016). Dietary 2-oxoglutarate mitigates gastrectomy-evoked structural changes in cartilage of female rats. Exp. Biol. Med..

[29] Dobrowolski P.J., Piersiak T., Surve V.V., Kruszewska D., Gawron A., Pacuska P., Håkanson R., Pierzynowski S.G. (2008). Dietary α-ketoglutarate reduces gastrectomy-evoked loss of calvaria and trabecular bone in female rats. Scand. J. Gastroenterol..

[30] Kim S.S., Christopher L., Bancroft J.D. (2019). Bancroft’s Theory and Practice of Histological Techniques.

[31] Rich L., Whittaker P. (2005). Collagen and Picrosirius Red Staining: A Polarized Light Assessment of Fibrillar Hue and Spatial Distribution. Braz. J. Morphol. Sci..

[32] Dobrowolski P., Huet P., Karlsson P., Eriksson S., Tomaszewska E., Gawron A., Pierzynowski S.G. (2012). Potato fiber protects the small intestinal wall against the toxic influence of acrylamide. Nutrition.

[33] Kisielinski K., Willis S., Prescher A., Klosterhalfen B., Schumpelick V. (2002). A simple new method to calculate small intestine absorptive surface in the rat. Clin. Exp. Med..

[34] Donaldson J., Świątkiewicz S., Arczewka-włosek A., Muszyński S., Szymań S. (2021). Modern hybrid rye as an alternative energy source for broiler chickens ameliorates absorption surface of initial segments of intestines regardless of xylanase supplementation. Animals.

[35] Seeley R.J., Chambers A.P., Sandoval D.A. (2015). The Role of Gut Adaptation in the Potent Effects of Multiple Bariatric Surgeries on Obesity and Diabetes. Cell Metab..

[36] Blachier F., Mariotti F., Huneau J.F., Tomé D. (2006). Effects of amino acid-derived luminal metabolites on the colonic epithelium and physiopathological consequences. Amino Acids.

[37] Eklou-Lawson M., Bernard F., Neveux N., Chaumontet C., Bos C., Davila-Gay A.-M., Tomé D., Cynober L., Blachier F. (2008). Colonic luminal ammonia and portal blood l-glutamine and l-arginine concentrations: A possible link between colon mucosa and liver ureagenesis. Amino Acids.

[38] Hou Y., Wang L., Ding B., Liu Y., Zhu H., Liu J., Li Y., Kang P., Yin Y., Wu G. (2011). Alpha-Ketoglutarate and intestinal function. Front. Biosci..

[39] Bergen W.G., Wu G. (2009). Intestinal Nitrogen Recycling and Utilization in Health and Disease. J. Nutr..

[40] Dokladny K., Zuhl M.N., Moseley P.L. (2016). Intestinal epithelial barrier function and tight junction proteins with heat and exercise. J. Appl. Physiol..

[41] He L., Zhou X., Huang N., Li H., Cui Z., Tian J., Jiang Q., Liu S., Wu J., Li T. (2017). Administration of alpha-ketoglutarate improves epithelial restitution under stress injury in early-weaning piglets. Oncotarget.

[42] Chou H.-C., Chen C.-M. (2017). Neonatal hyperoxia disrupts the intestinal barrier and impairs intestinal function in rats. Exp. Mol. Pathol..

[43] Lee B., Moon K.M., Kim C.Y. (2018). Tight Junction in the Intestinal Epithelium: Its Association with Diseases and Regulation by Phytochemicals. J. Immunol. Res..

[44] Steed E., Elbediwy A., Vacca B., Dupasquier S., Hemkemeyer S.A., Suddason T., Costa A.C., Beaudry J.-B., Zihni C., Gallagher E. (2014). MarvelD3 couples tight junctions to the MEKK1–JNK pathway to regulate cell behavior and survival. J. Cell Biol..

[45] Li X., Wang Q., Xu H., Tao L., Lu J., Cai L., Wang C. (2014). Somatostatin regulates tight junction proteins expression in colitis mice. Int. J. Clin. Exp. Pathol..

[46] Wang B., Wu Z., Ji Y., Sun K., Dai Z., Wu G. (2016). L-Glutamine Enhances Tight Junction Integrity by Activating CaMK Kinase 2–AMP-Activated Protein Kinase Signaling in Intestinal Porcine Epithelial Cells. J. Nutr..

[47] Zheng Y., Zhang M., Zhao Y., Chen J., Li B., Cai W. (2014). JNK inhibitor SP600125 protects against lipopolysaccharide-induced acute lung injury via upregulation of claudin-4. Exp. Ther. Med..

[48] Zdzisin B. (2016). Alpha-Ketoglutarate as a Molecule with Pleiotropic Activity: Well-Known and Novel Possibilities of Therapeutic Use. Arch. Immunol. Ther. Exp..

[49] Bayliak M.M., Lylyk M.P., Shmihel H.V., Sorochynska O.M., Semchyshyn O.I., Storey J.M., Storey K.B., Lushchak V.I. (2017). Dietary alpha-ketoglutarate promotes higher protein and lower triacylglyceride levels and induces oxidative stress in larvae and young adults but not in middle-aged Drosophila melanogaster. Comp. Biochem. Physiol. Part A Mol. Integr. Physiol..

[50] He L., Xu Z., Yao K., Wu G., Yin Y., Nyachoti C., Kim S. (2015). The Physiological Basis and Nutritional Function of Alpha-ketoglutarate. Curr. Protein Pept. Sci..

[51] SSzefel J., Kruszewski W.J., Buczek T. (2015). Enteral feeding and its impact on the gut immune system and intestinal mucosal barrier. Gastroenterol. Rev..

[52] Li B., Lu Y., Srikant C.B., Gao Z.-H., Liu J.-L. (2013). Intestinal adaptation and Reg gene expression induced by antidiabetic duodenal-jejunal bypass surgery in Zucker fatty rats. Am. J. Physiol. Liver Physiol..

[53] le Roux C.W., Borg C., Wallis K., Vincent R.P., Bueter M., Goodlad R., Ghatei M.A., Patel A., Bloom S.R., Aylwin S.J.B. (2010). Gut Hypertrophy after Gastric Bypass Is Associated With Increased Glucagon-Like Peptide 2 and Intestinal Crypt Cell Proliferation. Ann. Surg..

[54] TTaqi E., Wallace L.E., de Heuvel E., Chelikani P., Zheng H., Berthoud H.-R., Holst J.J., Sigalet D.L. (2010). The influence of nutrients, biliary-pancreatic secretions, and systemic trophic hormones on intestinal adaptation in a Roux-en-Y bypass model. J. Pediatr. Surg..

[55] McDuffie L.A., Bucher B.T., Erwin C.R., Wakeman D., White F.V., Warner B.W. (2011). Intestinal adaptation after small bowel resection in human infants. J. Pediatr. Surg..

[56] Fleming M.A., Ehsan L., Moore S.R., Levin D.E. (2020). The Enteric Nervous System and Its Emerging Role as a Therapeutic Target. Gastroenterol. Res. Pract..

[57] Bayliss W.M., Starling E.H. (1899). The movements and innervation of the small intestine. J. Physiol..

[58] Furness J.B. (2012). The enteric nervous system and neurogastroenterology. Nat. Rev. Gastroenterol. Hepatol..

[59] Ahlman H., Nilsson O. (2001). The gut as the largest endocrine organ in the body. Ann. Oncol..

[60] Edkins J.S. (1905). On the chemical mechanism of gastric secretion. Proc. R. Soc. Lond. Ser. B Contain. Pap. A Biol. Character.

[61] Grossman M.I. (1970). Gastrin, cholecystokinin, and secretin act on one receptor. Lancet.

[62] Ahlman H., Dahlström A. (1983). Vagal mechanisms controlling serotonin release from the gastrointestinal tract and pyloric motor function. J. Auton. Nerv. Syst..

[63] Chaudhri O., Small C., Bloom S. (2006). Gastrointestinal hormones regulating appetite. Philos. Trans. R. Soc. B Biol. Sci..

[64] Ducroc R., Guilmeau S., Akasbi K., Devaud H., Buyse M., Bado A. (2005). Luminal Leptin Induces Rapid Inhibition of Active Intestinal Absorption of Glucose Mediated by Sodium-Glucose Cotransporter 1. Diabetes.

[65] Maciejewski M.L., Arterburn D.E., Van Scoyoc L., Smith V.A., Yancy W.S., Weidenbacher H.J., Livingston E.H., Olsen M.K. (2016). Bariatric surgery and long-term durability of weight loss. JAMA Surg..

