# Supplementary material for: Dietary Alpha-Ketoglutarate Partially Abolishes Adverse Changes in the Small Intestine after Gastric Bypass Surgery in a Rat Model
Source: Nutrients. 2022 May 14;14(10):2062. doi: 10.3390/nu14102062 (PMC9146360; doi:10.3390/nu14102062)
Supplement: Supplementary file 1 [file nutrients-14-02062-s001.zip › nutrients-1645804-supplementary.pdf]

nutrients MDPI

Table S1. The effect of bariatric surgery and alpha-ketoglutarate administration on histomorphometry of selected parameters of rat duodenum.

Parameter	C	O	O+AKG	AKG
Number of undamaged villi/mm	1.1 ± 0.8[ab]	1.7 ± 1.4[a]	0.4 ± 0.3[b]	0.8 ± 0.9[b]
Number of damaged villi/mm	3.3 ± 0.6	3.8 ± 1.5	3.6 ± 1.7	3.8 ± 1.2
Number of open crypts/mm	5.5 ± 1.7[a]	3.2 ± 1.4[b]	2.8 ± 0.4[b]	3.7 ± 0.9[ab]
Number of closed crypts/mm	13.3 ± 1.6[ab]	10.1 ± 3.6[b]	15.6 ± 4.1[a]	17.2 ± 3.4[a]
Number of goblet cells per 100 μm of villi epithelium	2.0 ± 1.1	2.0 ± 0.9	1.8 ± 0.9	1.8 ± 1.0
Fractal dimension of the intestinal villi [D]	1.123 ± 0.040	1.123 ± 0.037	1.117 ± 0.029	1.111 ± 0.032
Fractal dimension of the villi epithelial surface [D]	1.765 ± 0.021[a]	1.747 ± 0.014[b]	1.769 ± 0.026[a]	1.768 ± 0.017[a]
Fractal dimension of the lacteal space [D]	1.672 ± 0.040	1.650 ± 0.037	1.666 ± 0.030	1.665 ± 0.043
Fractal dimension of the intestinal mucosa [D]	1.260 ± 0.134	1.207 ± 0.110	1.211 ± 0.113	1.212 ± 0.099
Number of Meissner's nerve ganglia/mm	1.3 ± 0.6	0.9 ± 0.3	0.4 ± 0.3	0.7 ± 0.6
Number of Auerbach nerve ganglia/mm	1.7 ± 0.6	1.5 ± 0.6	1.3 ± 0.5	1.2 ± 0.5
Number of Henle's nerve ganglia/mm	1.4 ± 0.5	1.0 ± 0.6	1.0 ± 0.8	0.7 ± 0.4
Total number of nerve ganglia/mm	4.4 ± 0.9	3.4 ± 1.1	2.8 ± 1.3	2.5 ± 1.3
The shape of nerve ganglia	0.6 ± 0.2	0.6 ± 0.2	0.6 ± 0.2	0.6 ± 0.2

Data are presented as mean ± SD. Different superscript letters in a row indicate significant differences between groups at p <0.05. C – control group undergoing sham surgery; O – group undergoing gastric bypass surgery; O + AKG – group undergoing gastric bypass surgery and receiving alpha-ketoglutarate (AKG); AKG – group undergoing sham surgery and receiving alpha-ketoglutarate. AKG was administered at a dose of 0.2g/kg/body weight/day in drinking water.

Table S2. The effect of bariatric surgery and alpha-ketoglutarate administration on histomorphometry of selected parameters of rat jejunum.

Parameter	C	O	O + AKG	AKG
Number of undamaged villi/mm	1.7 ± 1.1	1.5 ± 1.1	0.7 ± 0.7	1.4 ± 0.9
Number of damaged villi/mm	3.0 ± 1.0	3.4 ± 1.6	2.8 ± 0.6	4.0 ± 1.2
Number of open crypts/mm	3.4 ± 1.7	2.3 ± 1.4	2.2 ± 1.0	3.2 ± 0.8
Number of closed crypts/mm	13.4 ± 2.6[b]	8.8 ± 3.2[c]	18.4 ± 1.9[a]	17.8 ± 3.3[a]
Number of goblet cells per 100 μm of villi epithelium	2.6 ± 1.2	2.1 ± 0.8	2.1 ± 0.9	2.1 ± 0.9
Fractal dimension of the intestinal villi [D]	1.124 ± 0.052	1.110 ± 0.025	1.122 ± 0.043	1.111 ± 0.041
Fractal dimension of the villi epithelial surface [D]	1.759 ± 0.017	1.761 ± 0.022	1.753 ± 0.018	1.755 ± 0.024
Fractal dimension of the lacteal space [D]	1.652 ± 0.032[a]	1.649 ± 0.045[ab]	1.630 ± 0.033[b]	1.643 ± 0.040[ab]
Fractal dimension of the intestinal mucosa [D]	1.238 ± 0.156	1.180 ± 0.056	1.237 ± 0.103	1.214 ± 0.071
Number of Meissner's nerve ganglia/mm	0.4 ± 0.3	0.4 ± 0.1	0.3 ± 0.2	0.4 ± 0.2
Number of Auerbach nerve ganglia/mm	1.0 ± 0.5	1.0 ± 0.3	1.1 ± 0.4	0.9 ± 0.3
Number of Henle's nerve ganglia/mm	1.6 ± 0.3	1.8 ± 0.3	1.4 ± 0.5	1.1 ± 0.6
Total number of nerve ganglia/mm	3.0 ± 0.8	3.2 ± 0.1	2.8 ± 0.8	2.4 ± 0.9
The shape of nerve ganglia	0.6 ± 0.2	0.6 ± 0.2	0.6 ± 0.2	0.6 ± 0.2

Data are presented as mean ± SD. Different superscript letters in a row indicate significant differences between groups at p <0.05. C – control group undergoing sham surgery; O – group undergoing gastric bypass surgery; O+AKG – group undergoing gastric bypass surgery and receiving alpha-ketoglutarate (AKG); AKG – group undergoing sham surgery and receiving alpha-ketoglutarate. AKG was administered at a dose of 0.2g/kg/body weight/day in drinking water.
